# Building equitable health partnerships: addressing racial disparities in global health

**DOI:** 10.3389/fpubh.2025.1604892

**Published:** 2025-05-26

**Authors:** Bruno Bonnechère

**Affiliations:** ^1^REVAL Rehabilitation Research Center, Faculty of Rehabilitation Sciences, Hasselt University, Diepenbeek, Belgium; ^2^Technology-Supported and Data-Driven Rehabilitation, Data Sciences Institute, Hasselt University, Diepenbeek, Belgium; ^3^Department of PXL – Healthcare, PXL University of Applied Sciences and Arts, Hasselt, Belgium

**Keywords:** health inequalities, racial and ethnic differences, global health, cooperation, health disparities

## Abstract

The traditional paradigms in global health, often characterized by power imbalances similar to the racial disparities between White and Black populations, are insufficient for addressing the complex health challenges of the 21st century. These disparities not only exist within national borders but also mirror the limitations of the North–South paradigm on an international scale. This framework perpetuates systemic inequalities, undermines local agency, and neglects the valuable expertise within communities of color. The evolving landscape of global health, marked by emerging infectious diseases, antimicrobial resistance, non-communicable diseases, and climate change impacts, necessitates a paradigm shift toward partnerships based on mutual respect, shared responsibility, and equitable collaboration. This paper explores the limitations of the conventional paradigms and highlights the multifaceted benefits of a more collaborative approach. It demonstrates how equitable partnerships can enhance health security, foster innovation, and promote sustainable development across racial lines. Successful examples of equity-focused cooperation illustrate the potential of diverse partnerships in strengthening health systems and promoting knowledge sharing between White and Black communities. A new framework for health cooperation is proposed, emphasizing mutual respect, transparency, accountability, and sustainable capacity building. By recognizing the agency and expertise of Black communities, we can create a more inclusive and democratic health architecture. This shift from a charity-based mindset to one rooted in solidarity acknowledges that investing in health equity is a strategic investment in our collective future. Embracing this interconnected approach will enable us to tackle pressing racial health challenges and ensure a healthier and more equitable future for all.

## 1 Introduction

Health disparities between White and Black populations have long been evident in the global health landscape. International cooperation in health has indeed long been viewed through the lens of a North–South divide, a paradigm where developed nations (the “North” – or the “White”) provide aid and expertise to developing countries (the “South” – or the “Black”) (1). This framework, while acknowledging historical imbalances and genuine needs, has increasingly become inadequate for addressing the complex and interconnected challenges of the 21st century (2). It often perpetuates a donor-recipient dynamic, overlooking the agency and potential contributions of developing countries and failing to recognize the shared vulnerabilities all nations face in an era of globalization. While the need for development assistance remains, the traditional aid-based model risks hindering true collaboration and innovation, impeding progress toward addressing the structural inequities affecting Black populations, ultimately delaying progress toward a healthier and more secure future for all (3).

This paper argues that a paradigm shift is essential. We must move beyond the limited scope of traditional frameworks and embrace a new vision of cooperation in health – one built on mutual respect, shared responsibility, and a recognition of the interconnectedness of health disparities. The evolving landscape of health threats, from emerging infectious diseases and antimicrobial resistance to the growing burden of non-communicable diseases and the impact of climate change, transcends racial boundaries (4, 5). The intersectionality of social conditions such as poverty and marginality with racial issues exacerbates health disparities (6). Understanding and addressing these intersecting factors is crucial for developing effective and equitable health interventions. This requires a holistic approach that considers the multifaceted nature of health inequities (7). No single community, regardless of its economic or political standing, can effectively address these challenges in isolation. This necessitates a move away from traditional models toward genuine partnerships where all communities, regardless of their racial background, contribute their unique strengths and expertise.

We will first explore the limitations of existing paradigms, highlighting how they hinder progress and perpetuate racial inequalities. We will then delve into the multifaceted benefits of a more collaborative and equitable approach, demonstrating how such partnerships can enhance health security, foster innovation, and promote sustainable racial equity. Through case studies of successful equity-focused cooperation, we will showcase the potential of diverse partnerships and highlight best practices. Finally, we will propose a new framework for health cooperation, emphasizing the roles of various stakeholders and outlining concrete steps toward a more just and effective health architecture. Ultimately, this paper aims to contribute to a reframing of the health narrative, emphasizing the crucial role of genuine partnership for achieving a healthier and more equitable future for all.

## 2 The evolving landscape of global health

The landscape of global health is in constant flux, characterized by increasingly complex and interconnected challenges (8). The 21st century has witnessed the emergence and re-emergence of infectious diseases, such as SARS (9), Ebola (10), Zika (11), and most recently, COVID-19 (12), demonstrating the rapid and often unpredictable nature of these threats. These outbreaks underscore the porous nature of borders and the vulnerability of even the most advanced healthcare systems to unforeseen pathogens. Beyond infectious diseases, the rise of antimicrobial resistance (AMR) poses a grave threat to modern medicine, potentially rendering many life-saving treatments ineffective (13). The increasing prevalence of AMR, often driven by inappropriate antibiotic use in both human and animal health, requires a concerted global effort to develop new antibiotics, promote responsible use, and strengthen surveillance systems (14).

Simultaneously, the global burden of non-communicable diseases (NCDs) like cardiovascular disease, cancer, diabetes, and chronic respiratory conditions continues to rise (15), particularly in low-and middle-income countries (LMICs) and particularly in Africa (16). But these disparities go well behind the geographical localities, it has been clearly illustrated that Black people, regardless of their country of residence, have higher risk of hypertension (17) and diabetes (18), two of the most important modifiable risk factor of NCSs (19).

These diseases often require long-term management and place a significant strain on health systems worldwide. Addressing the growing NCD burden requires a multi-sectoral approach that encompasses prevention, early detection, and affordable access to quality care (20). Furthermore, climate change acts as a threat multiplier, exacerbating existing health challenges and creating new ones. Rising temperatures, extreme weather events, and changes in vector ecology are impacting infectious disease transmission patterns, food security, and access to clean water and sanitation, disproportionately affecting vulnerable populations (21).

These evolving health challenges highlight the interconnectedness of our world and the shared vulnerabilities faced by all nations, but most importantly between the nations according to the different background and ethnicities. There are numerous examples across developed, or high-income countries, of huge discrepancies between White and Black people (22). Disparities in life expectancy among racial-ethnic groups are widespread and enduring in the states with an average difference of 5 years (23). Health disparities are worsened when social factors like poverty and marginalization intersect with racial issues (24). Effective and equitable health interventions depend on recognizing and tackling these factors. This necessitates a comprehensive strategy that acknowledges the complex interplay of elements contributing to health inequities.

The consideration of mental health is also pivotal, especially as it constitutes one of the biggest causes of health inequity and inequality, notably for Black populations in the global North (25). The disproportionate impact of mental health disorders among Black communities underscores the need for tailored interventions that address both the psychological and socio-economic factors contributing to these disparities (26). Addressing mental health inequities requires a comprehensive approach that integrates culturally sensitive mental health services with broader health initiatives aimed at reducing overall health disparities.

The traditional focus on isolated health issues within specific geographical boundaries is no longer sufficient. The COVID-19 pandemic starkly demonstrated how quickly a health crisis in one part of the world can escalate into a global emergency, impacting not only health systems but also economies, social structures, and political stability (27). Here also huge disparities have been found between the diagnosis, management, and survival rate between White and Black (28–30). While racial disparities have been highlighted, it is equally important to consider all ethnic minorities and conditions of social vulnerability, as revealed during the COVID-19 pandemic. The pandemic illuminated the compounded challenges faced by marginalized groups, underscoring the necessity for an inclusive approach that addresses the diverse needs of these communities in health policies and programs (31). Therefore, moving away from disease-specific interventions should be linked to refocusing on social determinants of health. Addressing the underlying social and economic factors that contribute to health disparities is essential for achieving sustainable improvements in population health (32). This requires an integrated approach that links health and social care, recognizing the interconnected nature of these domains.

Another significant factor contributing to health inequities is access to care. Structural barriers, including socio-economic status, geographic location, and systemic racism, restrict access to quality health care for many minority populations (33). Ensuring equitable access to care is crucial for mitigating the health disparities that disproportionately affect these groups.

These challenges necessitate a shift away from fragmented and reactive approaches toward a more proactive, collaborative, and holistic approach to global health. We need a paradigm shift that recognizes the shared responsibility of all nations in safeguarding global health security and promoting well-being for all. This requires moving beyond the traditional aid-based model and fostering genuine partnerships based on mutual respect, shared learning, and joint action.

## 3 Limitations of North–South paradigm in health

The traditional North–South paradigm in global health, while instrumental in addressing certain health challenges, suffers from inherent limitations that hinder its effectiveness in the face of evolving global health realities (34). This framework, characterized by a predominantly unidirectional flow of resources and expertise from developed countries (*the “North”*) to developing countries (*the “South”*), often perpetuates power imbalances and reinforces dependencies (35), same inadequate unidirectional direction is also very often see at national levels between White and Black people (36). While well-intentioned, this approach can undermine local ownership and agency, hindering the development of sustainable and contextually appropriate health solutions.

One of the key criticisms of the North–South paradigm is its inherent asymmetry. Decision-making power often resides primarily with donor countries and international organizations, while recipient countries are relegated to the role of implementers (37). This power dynamic can lead to a disconnect between program design and local needs, resulting in interventions that are not culturally sensitive or sustainable in the long term (38). Furthermore, the focus on vertical programs targeting specific diseases often neglects the strengthening of overall health systems, which are crucial for addressing a wide range of health challenges (39). This can create parallel systems and further fragment already fragile health infrastructures (40).

The North–South paradigm also often overlooks the valuable knowledge and expertise that exist within developing countries. Local communities possess a deep understanding of their own health needs and priorities, as well as innovative solutions tailored to their specific contexts. By failing to adequately engage local stakeholders in the design and implementation of health interventions, the North–South approach misses opportunities for valuable learning and collaboration. This can result in missed opportunities for innovation and the development of more effective and sustainable health solutions (41).

Furthermore, the traditional focus on aid can create a cycle of dependency, where recipient countries become reliant on external funding and expertise (42). This can hinder the development of local capacity and discourage investment in domestic health systems. The emphasis on quantifiable outputs and short-term impact, often driven by donor priorities, can also detract from long-term sustainable development goals. This can lead to a focus on easily measurable outcomes, neglecting the more complex and nuanced aspects of health system strengthening and community empowerment (43).

Finally, the North–South paradigm often fails to address the underlying determinants of health, such as poverty, inequality, and social injustice (24). While providing essential health services is crucial, addressing these root causes is essential for achieving sustainable improvements in population health. A narrow focus on disease-specific interventions without addressing these broader societal factors can limit the long-term impact of health programs and perpetuate health disparities.

In conclusion, while the North–South paradigm has played a role in addressing certain health challenges, its inherent limitations are increasingly apparent in the face of evolving global health threats. The power imbalances, dependency dynamics, and neglect of local expertise inherent in this framework hinder the development of sustainable and equitable health solutions. A paradigm shift toward more collaborative and mutually beneficial partnerships is essential for achieving global health security and ensuring health for all. Furthermore, as we have already seen, access to care is a major factor contributing to health disparities. Ensuring equitable access to quality healthcare services is thus essential for addressing the health challenges faced by marginalized communities. This requires a concerted effort to remove barriers to care and promote inclusive health systems.

## 4 Mutual benefit of reframing global cooperation

Reframing global health cooperation beyond the traditional North–South paradigm unlocks a wealth of mutual benefits for both developed and developing countries. Embracing a model grounded in partnership, shared responsibility, and mutual learning creates a more effective and sustainable approach to addressing global health challenges.

### 4.1 Benefit for developed countries

#### 4.1.1 Access to diverse perspectives and innovative solutions

Developing countries offer a wealth of untapped knowledge and experience in tackling unique health challenges with limited resources. This ingenuity can lead to innovative solutions applicable in diverse settings, including resource-constrained environments within developed countries. Collaborations can expose developed nations to novel approaches to community health worker programs, telemedicine applications, and low-cost diagnostic tools, potentially enhancing their own health systems’ efficiency and reach.

#### 4.1.2 Enhanced research and development capabilities

Partnerships with developing countries provide access to diverse populations and disease burdens, enriching research opportunities and accelerating the development of new diagnostics, treatments, and vaccines. Conducting clinical trials and epidemiological studies in diverse settings generates more robust and generalizable data, leading to more effective interventions for all populations. Collaborative research also fosters scientific capacity building in developing countries, strengthening global health research infrastructure.

#### 4.1.3 Improved global health security and reduced risk of cross-border health threats

Infectious diseases know no borders. Investing in health systems strengthening in developing countries is a direct investment in global health security. Stronger health systems are better equipped to detect, contain, and respond to outbreaks at their source, preventing them from escalating into global pandemics that threaten all nations. Collaborative surveillance systems, information sharing, and joint training exercises enhance preparedness and response capabilities worldwide.

#### 4.1.4 Strengthened diplomatic ties and global influence

Engaging in genuine partnerships fosters trust and mutual understanding between nations. Health diplomacy, built on shared health goals, can bridge political divides and strengthen international relations. By demonstrating a commitment to global health solidarity, developed countries can enhance their global standing and influence.

### 4.2 Benefits for developing countries

#### 4.2.1 Increased ownership and agency in shaping health interventions

Moving away from the donor-recipient dynamic empowers developing countries to take the lead in identifying their own health priorities and designing interventions tailored to their specific contexts. This ensures that programs are culturally appropriate, sustainable, and aligned with national development goals. Increased ownership fosters accountability and strengthens local commitment to program success.

#### 4.2.2 Enhanced capacity building and sustainable health systems strengthening

True partnerships prioritize capacity building within developing countries, fostering self-reliance and long-term sustainability. This involves investing in health workforce development, strengthening health infrastructure, and improving data collection and analysis capabilities. By focusing on long-term capacity building rather than short-term project deliverables, collaboration can lead to more resilient and responsive health systems.

#### 4.2.3 Improved access to essential health technologies and resources

Partnerships can facilitate access to essential medicines, vaccines, diagnostics, and other health technologies. This can involve technology transfer, joint manufacturing agreements, and innovative financing mechanisms. Equitable access to these resources is crucial for addressing health disparities and ensuring that everyone has access to the healthcare they need.

#### 4.2.4 Greater equity in global health governance

A more inclusive approach to global health governance ensures that the voices of developing countries are heard and their priorities are considered in international decision-making processes. This requires reforming existing global health institutions to ensure greater representation and participation from all countries, fostering a more equitable and democratic global health architecture.

In conclusion, moving beyond the traditional North–South paradigm creates a win-win scenario for all countries. By embracing a truly collaborative approach to global health, we can unlock the full potential of global partnerships, accelerate progress toward achieving health for all, and build a safer and more equitable future for everyone.

## 5 Successful examples of South–South and triangular cooperation

**Figure 1:**
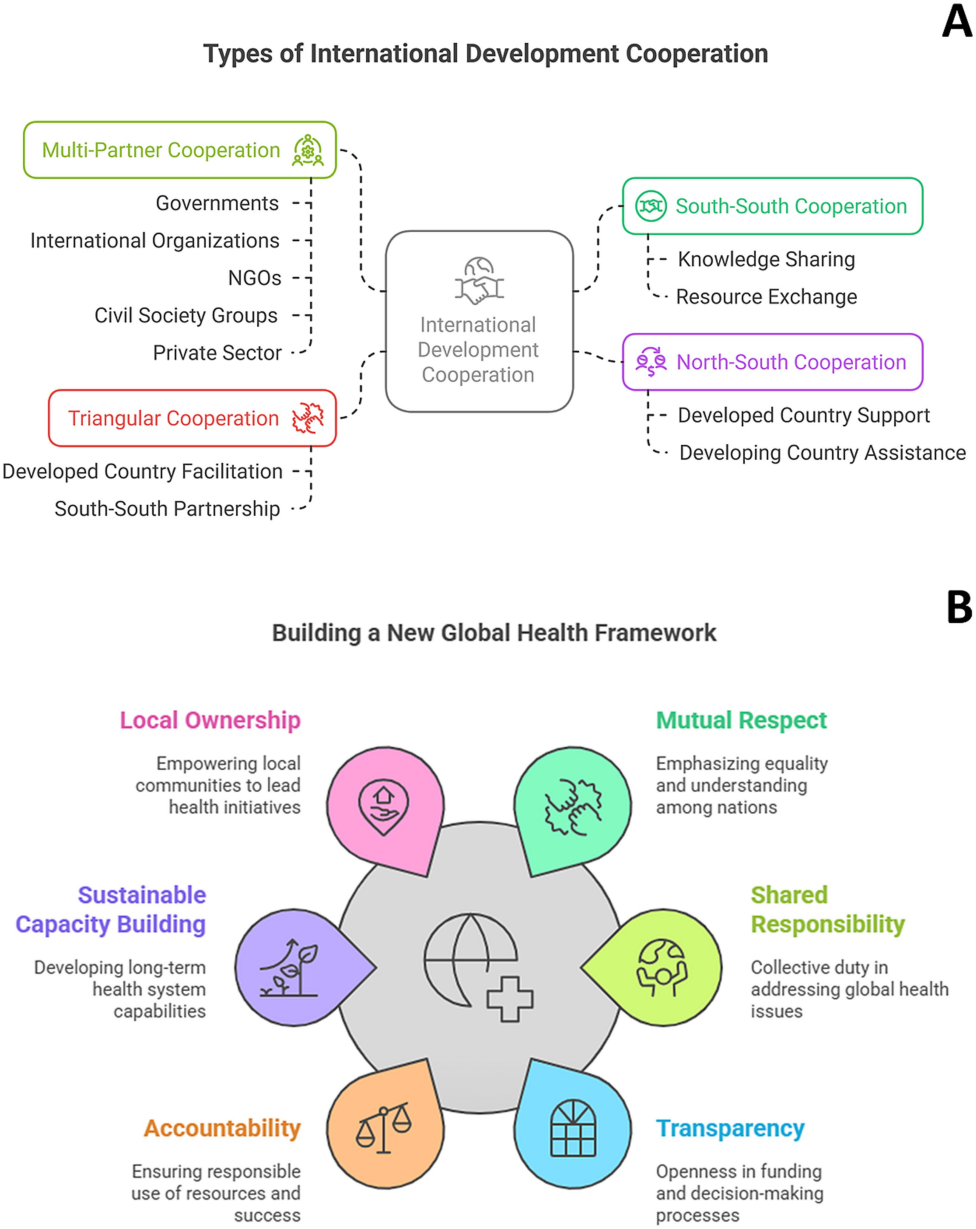
**(A)** The different models of international development cooperation. **(B)** A more equitable and transparent framework for cooperation in a global world supported by six key principles. The circular arrangement underscores the interconnectedness of these principles. This framework prioritizes equitable partnerships and access to resources to effectively address global health challenges.

Different models of international collaboration exist, each characterized by distinct partnerships and resource flows (Figure 1A). South–South cooperation involves collaborations primarily between developing countries, fostering knowledge, resource, and expertise sharing without significant developed nation involvement (44). North–South cooperation represents the traditional development assistance model, with a developed country providing support to a developing country (45). Triangular cooperation incorporates a partnership between two developing countries (South–South) facilitated and/or supported by a developed country (46). Finally, multi-partner cooperation signifies collaborations encompassing a diverse range of actors, including governments from both developed and developing countries, international organizations, NGOs, civil society groups, and sometimes the private sector (47).

Examining successful instances of South–South and triangular cooperation provides concrete evidence of the potential of collaborative partnerships in addressing global health challenges. These examples demonstrate the effectiveness of moving beyond traditional donor-recipient dynamics and fostering mutual learning and shared responsibility. These case studies, presented in the Table 1, offer valuable insights into the diverse forms that international health cooperation can take. While numerous projects address global health, these three diverse examples highlight different approaches: two initiatives focus on distinct vulnerable populations, while a third emphasizes regional collaboration. The Triangular Cooperation for Maternal and Child Health in Africa leverages the expertise of Japan and Brazil to adapt and implement community health worker programs in various African countries, focusing on training, material development, and system strengthening (48). WHO Rehabilitation 2030 (49) and the World Rehabilitation Alliance (50) operate on a global scale, advocating for strengthened rehabilitation services through policy development and capacity building for people with disabilities. Meanwhile, the East, Central, and Southern Africa Health Community (ECSA-HC) represents a regional approach (51). This intergovernmental organization works with 16 member states to address shared health challenges like disease control and health information systems strengthening through joint planning and resource mobilization, while navigating obstacles such as political instability and resource constraints. Each initiative, though different in scope and approach, contributes to a stronger global health landscape. These diverse examples demonstrate the flexibility and adaptability of collaborative partnerships in tackling a wide range of global health challenges, offering valuable lessons for future collaborations.

**Table 1:** Overview of a few global health programs showing the different levels of cooperation.

Project	Focus area	Brief summary	Key partners	Cooperation dimension	Target beneficiaries	Key activities	Challenges
International Atomic Energy Agency (IAEA) – supporting cancer control in developing countries	Cancer Control	This initiative provides essential support for cancer control in developing countries, focusing on the use of radiotherapy, nuclear medicine, and other nuclear techniques. The goal is to enhance cancer treatment capabilities and improve patient outcomes.	IAEA, various developing countries	North–South and Triangular (often involving partnerships with other developed countries)	Patients in developing countries requiring cancer diagnosis and treatment	Training healthcare professionals, providing equipment, developing treatment guidelines, supporting research.	Limited resources, infrastructure gaps, workforce shortages, sustainability of programs
South African Development Community (SADC) Pharmaceutical Programme (56)	Access to Medicines and Pharmaceutical Services	Aims to improve access to medicines and pharmaceutical services in the Southern African Development Community region through joint procurement, regulatory harmonization, and capacity building.	SADC Member States, development partners (e.g., pharmaceutical companies, WHO, Global Fund)	South–South and North–South	Populations of SADC member states	Joint procurement of essential medicines, harmonization of drug registration procedures, strengthening pharmaceutical manufacturing capacity.	Varying levels of development among member states, logistical challenges, limited manufacturing capacity in the region
Triangular Cooperation for Maternal and Child Health in Africa (48)	Maternal and Child Health	Adapting and implementing community health worker programs to improve maternal and child health outcomes.	Japan, Brazil, Various African countries	Triangular	Mothers and children in African countries	Training community health workers, developing educational materials, strengthening community health systems.	Context-specific challenges, ensuring sustainability, coordination among partners
SEAMEO TROPMED Regional Centre for Public Health, Nutrition and Food Security (57)	Public Health, Nutrition, Food Security	Addresses nutrition and food security concerns with collaborative projects and research at a regional level.	Primarily Southeast Asian countries, but includes partnerships with institutions in other regions (e.g., Australia, US)	South–South and Triangular	Populations of Southeast Asian countries	Research, training, policy development, information sharing.	Resource mobilization, varying capacities among member states
East, Central, and Southern Africa Health Community (ECSA-HC) (51)	Regional Health Cooperation	Intergovernmental health organization addressing regional health challenges through joint planning, resource mobilization, and capacity building.	16 countries in East, Central, and Southern Africa	South–South	Populations of ECSA-HC member states	Developing regional health strategies, coordinating disease control programs, strengthening health information systems.	Political instability in some member states, resource constraints, varying health systems
Elimination 8 Initiative for Polio Eradication (58)	Polio Eradication	Focused effort to eradicate polio in eight African countries through synchronized vaccination campaigns and surveillance.	Governments of the different countries, WHO, UNICEF, Rotary International, CDC, Bill and Melinda Gates Foundation	Multi-Partner (including South–South and North–South elements)	Children in eight African countries	Synchronized vaccination campaigns, surveillance, community mobilization.	Reaching marginalized communities, insecurity in some areas, maintaining political commitment
Global Fund to Fight AIDS, Tuberculosis and Malaria (59)	HIV/AIDS, Tuberculosis, Malaria	Partnership to accelerate the end of AIDS, tuberculosis and malaria as epidemics by providing funding and technical support to countries.	Governments, civil society, technical agencies, the private sector, and people affected by the diseases	Multi-Partner	People affected by HIV/AIDS, tuberculosis, and malaria	Grant funding, technical assistance, capacity building.	Ensuring sustainable financing, addressing drug resistance
Pandemic Influenza Preparedness Framework (PIP Framework) (60)	Pandemic Preparedness	Promotes the sharing of influenza viruses with human pandemic potential and benefits arising from such sharing, including access to vaccines and other benefits.	WHO Member States, other stakeholders (e.g., vaccine manufacturers, research institutions)	Multi-Partner	Global population	Virus sharing, research and development, capacity building.	Ensuring equitable benefit sharing, navigating complex international agreements
WHO Rehabilitation 2030 (49) and the World Rehabilitation Alliance (50)	Rehabilitation Services	Global strategy to strengthen and expand rehabilitation services worldwide through advocacy, policy development, and capacity building.	Multi-Partner (facilitates various collaborations at different levels)	Multi-Partner	People with disabilities and other health conditions requiring rehabilitation	Advocacy, policy development, technical assistance, research.	Raising awareness about the importance of rehabilitation, integrating rehabilitation into health systems

## 6 Toward a new framework for global health cooperation

Moving beyond the limitations of the North–South paradigm requires a fundamental shift in how we conceptualize and practice international cooperation in health. A new framework must be built on principles of mutual respect, shared responsibility, transparency, and accountability, prioritizing sustainable capacity building, local ownership, and equitable access to health resources and technologies. This framework should move away from traditional donor-recipient relationships toward equitable partnerships where all countries have a voice and contribute their unique strengths. Recognizing the agency and expertise of developing countries is crucial, ensuring their priorities are reflected in global health agendas (52). This necessitates embracing shared responsibility, acknowledging that global health is a collective concern requiring action from all nations, regardless of their development status. Contributions should extend beyond financial assistance to encompass sharing knowledge, technology, and best practices (53).

As presented in Figure 1B, transparency and accountability are cornerstones of this new framework. This involves promoting transparency in funding, decision-making, and program implementation, coupled with establishing clear mechanisms for accountability to ensure effective resource utilization and program success. Sustainable capacity building within developing countries is paramount, fostering self-reliance and long-term health system strengthening. Investments in health workforce development, infrastructure improvements, and enhanced data capabilities are crucial components of this process. Furthermore, ensuring local ownership is essential for effective and sustainable interventions. This means designing and implementing programs in partnership with local communities and governments, respecting local knowledge, cultural contexts, and priorities.

Equitable access to health resources and technologies is another critical element. Promoting access to essential medicines, vaccines, diagnostics, and other health technologies requires exploring innovative financing mechanisms, technology transfer, and joint manufacturing agreements to guarantee affordability and availability for all. Finally, strengthening multilateralism is vital for effective global health governance. This involves reinforcing the role of international organizations like the WHO in coordinating global health efforts and providing technical assistance (54). Critically, reforming global health governance is necessary to ensure greater representation and participation from developing countries, creating a more equitable and democratic global health architecture. This multifaceted approach necessitates a shift from a charity-based mindset to one rooted in solidarity, recognizing that investing in global health is not merely an act of benevolence but a strategic investment in our collective future (55). By embracing these interconnected principles, we can forge a more just, effective, and sustainable approach to global health cooperation, paving the way for a healthier and more secure future for all.

## 7 Conclusion

As we navigate the intricate landscape of global health in the 21st century, it is evident that the conventional North–South paradigm falls short in addressing the multifaceted challenges we face today. The inherent limitations of this model—characterized by power imbalances, dependency dynamics, and a narrow focus on aid—hinder the development of sustainable and equitable health solutions. It is imperative that we move beyond these outdated frameworks and embrace a transformative vision of global health cooperation. Building a caring community is intrinsically linked to policies based on local democracy and service networking. Including human resources such as community health and social workers is essential for creating a supportive and inclusive health environment. This approach ensures that local needs and priorities are fully addressed, fostering a sense of community ownership and responsibility guarantying sustainable implementation of such initiatives.

By reframing our approach, we unlock a multitude of mutual benefits for both developed and developing nations. Collaborations rooted in partnership, shared responsibility, and mutual learning enable us to harness the diverse perspectives and innovative solutions that arise from different contexts. Such partnerships enhance research and development capabilities, improve global health security, and strengthen diplomatic ties, creating a robust foundation for tackling shared health threats.

Successful examples of South–South and triangular cooperation illustrate the potential of collaborative partnerships in fostering health security, strengthening health systems, and promoting knowledge sharing across regions. These case studies underscore the importance of moving away from traditional donor-recipient dynamics toward genuine partnerships that prioritize sustainable capacity building, local ownership, and equitable access to health resources. Moving away from disease-specific interventions and refocusing on social determinants of health is also crucial for addressing the underlying causes of health disparities. This integrated approach, linking health and social care, is essential for achieving sustainable improvements in population health and ensuring a healthier future for all.

The path forward is clear: we must adopt a new framework for global health cooperation. This framework should be built on principles of mutual respect, transparency, and accountability, with a focus on sustainable capacity building and equitable access to health resources. By recognizing the expertise and agency of developing countries, we can create a more inclusive and democratic global health architecture, where all voices are heard, and diverse contributions are valued.

In doing so, we transition from a charity-based mindset to one rooted in solidarity, acknowledging that investing in global health is not only an act of benevolence but a strategic investment in our collective future. Embracing this interconnected and equitable approach to global health cooperation will enable us to tackle the pressing health challenges of our time and pave the way for a healthier and more secure future for all. The imperative for genuine partnership is clear: our global health security depends on it.

## Data Availability

The original contributions presented in the study are included in the article/supplementary material, further inquiries can be directed to the corresponding author.
